# An overview of the use of bacteriophages in the poultry industry: Successes, challenges, and possibilities for overcoming breakdowns

**DOI:** 10.3389/fmicb.2023.1136638

**Published:** 2023-03-21

**Authors:** Amr Abd-El Wahab, Shereen Basiouni, Hesham R. El-Seedi, Marwa F. E. Ahmed, Lisa R. Bielke, Billy Hargis, Guillermo Tellez-Isaias, Wolfgang Eisenreich, Hansjörg Lehnherr, Sophie Kittler, Awad A. Shehata, Christian Visscher

**Affiliations:** ^1^Institute for Animal Nutrition, University of Veterinary Medicine Hannover Foundation, Hannover, Germany; ^2^Department of Nutrition and Nutritional Deficiency Diseases, Faculty of Veterinary Medicine, Mansoura University, Mansoura, Egypt; ^3^Cilia Cell Biology, Institute of Molecular Physiology, Johannes-Gutenberg University, Mainz, Germany; ^4^Clinical Pathology Department, Faculty of Veterinary Medicine, Benha University, Moshtohor, Toukh, Egypt; ^5^International Research Center for Food Nutrition and Safety, Jiangsu University, Zhenjiang, China; ^6^International Joint Research Laboratory of Intelligent Agriculture and Agri-Products Processing, Jiangsu Education Department, Jiangsu University, Nanjing, China; ^7^Department of Chemistry, Faculty of Science, Menoufia University, Shebeen El-Kom, Egypt; ^8^Department of Hygiene and Zoonoses, Faculty of Veterinary Medicine, Mansoura University, Mansoura, Egypt; ^9^Department of Animal Sciences, The Ohio State University, Columbus, OH, United States; ^10^Division of Agriculture, Department of Poultry Science, University of Arkansas, Fayetteville, AR, United States; ^11^Structural Membrane Biochemistry, Bavarian NMR Center, Technical University of Munich (TUM), Garching, Germany; ^12^PTC Phage Technology Center GmbH, a Part of Finktec Group, Bönen, Germany; ^13^Institute for Food Quality and Food Safety, University of Veterinary Medicine Hannover Foundation, Hannover, Germany; ^14^Avian and Rabbit Diseases Department, Faculty of Veterinary Medicine, University of Sadat City, Sadat City, Egypt; ^15^Research and Development Section, PerNaturam GmbH, An der Trift, Gödenroth, Germany; ^16^Prophy-Institute for Applied Prophylaxis, Bönen, Germany

**Keywords:** bacteriophages, challenges, alternative antimicrobials, poultry, bacteria

## Abstract

The primary contaminants in poultry are *Salmonella enterica*, *Campylobacter* j*ejun*i, *Escherichia coli,* and *Staphylococcus aureus*. Their pathogenicity together with the widespread of these bacteria, contributes to many economic losses and poses a threat to public health. With the increasing prevalence of bacterial pathogens being resistant to most conventional antibiotics, scientists have rekindled interest in using bacteriophages as antimicrobial agents. Bacteriophage treatments have also been investigated as an alternative to antibiotics in the poultry industry. Bacteriophages’ high specificity may allow them only to target a specific bacterial pathogen in the infected animal. However, a tailor-made sophisticated cocktail of different bacteriophages could broaden their antibacterial activity in typical situations with multiple clinical strains infections. Bacteriophages may not only be used in terms of reducing bacterial contamination in animals but also, under industrial conditions, they can be used as safe disinfectants to reduce contamination on food-contact surfaces or poultry carcasses. Nevertheless, bacteriophage therapies have not been developed sufficiently for widespread use. Problems with resistance, safety, specificity, and long-term stability must be addressed in particular. This review highlights the benefits, challenges, and current limitations of bacteriophage applications in the poultry industry.

## 1. Introduction

The application of antibiotics on poultry farms has been linked to the global emergence of multi-drug-resistant (MDR) bacteria in recent years (Benrabia et al., 2020; Kaonga et al., 2021). MDR bacteria can spread from food-producing animals to humans through direct contact with the food chain and the environment (Kirbis and Krizman, 2015). One-Health issues are still an important topic in the poultry industry, pre-harvest as well as post-harvest. Efforts must be made to reduce food-borne pathogen infection and/or contamination of the live birds before their dispatch to processing plants (Hafez, 1999). Post-harvest practices mainly rely on the use of physical and chemical approaches, which are not always successful in reducing pathogen load because of various factors like limited practicable concentration/temperature. In this context, finding more effective alternative antimicrobials is crucial to combatting risks to poultry production by targeting pathogens selectively without disturbing the microbiota and mitigating contamination of the food chain with food-borne pathogens.

**Figure 1:**
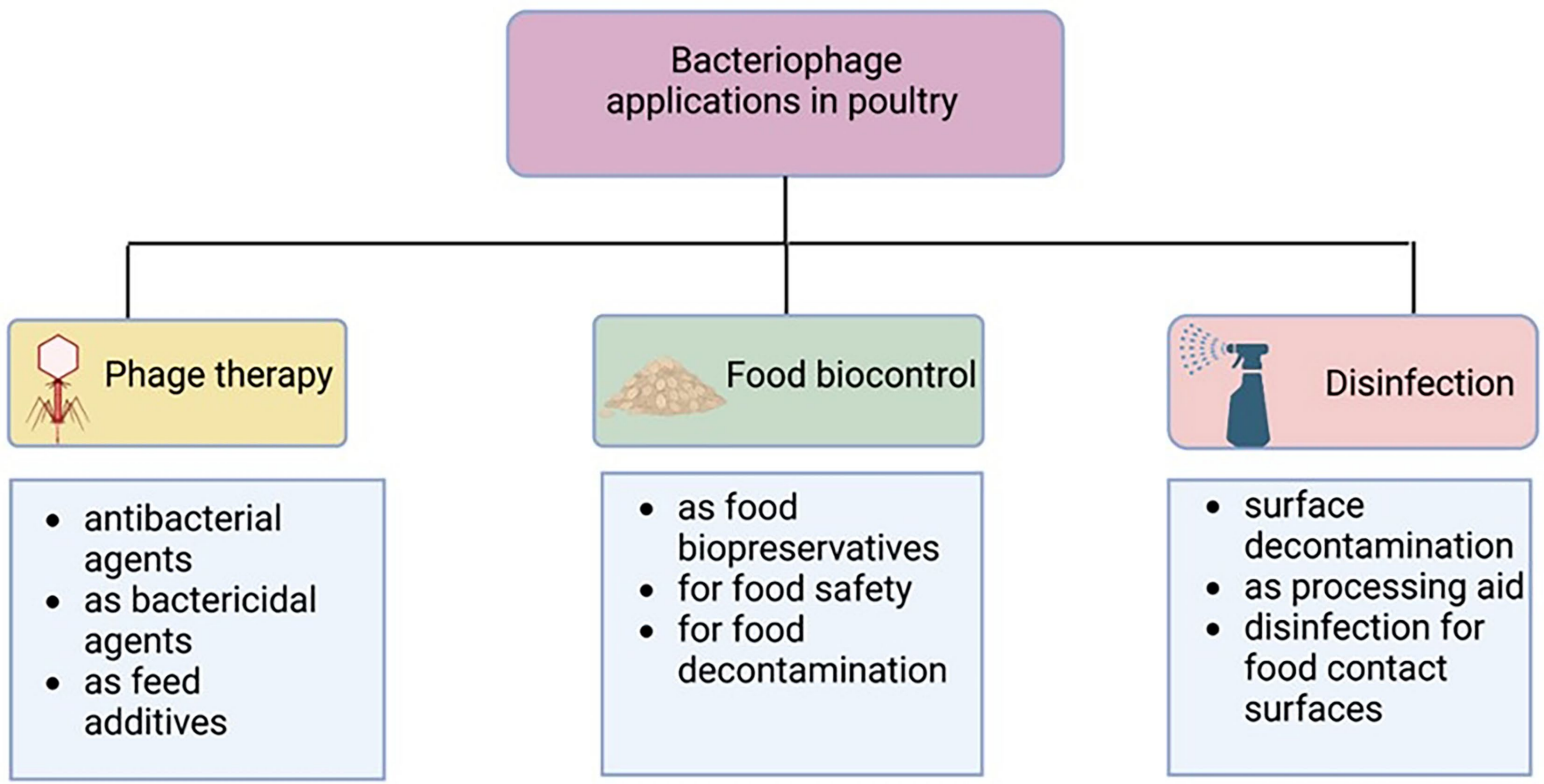
Bacteriophage uses in poultry industry. Created with BioRender.com.

The use of bacteriophage as therapeutics (phage therapy) may help to cope with the burden of MDR and may be considered an important alternative to antibiotics in the poultry industry (Wernicki et al., 2017; Moye et al., 2018). Figure 1 displays instances of phage functions in poultry production. Although several encouraging accounts of the use of bacteriophages in poultry have been documented, some negative results have also been reported (Loc-Carrillo and Abedon, 2011). This review focuses on the challenges and opportunities of the use of bacteriophages in the poultry industry.

## 2. Bacteriophages

### 2.1. Phages in nature

Bacteriophages, which literally means bacteria eaters, are viruses that selectively target and infect bacteria. Twort (1915) and D'herelle (1917) independently discovered bacteriophages, respectively (Duckworth, 1976). Bacteriophages are the most abundant organisms on earth. Gómez-Gómez et al. (2019) estimated that around 4.8 × 10^31^ phage particles are thought to be present in the biosphere overall, which is 10 times higher than the number of bacterial cells on earth. Bacteriophages, like other viruses, require a host cell to replicate. The majority of phages are highly specific and can only infect a limited range of closely related bacteria (de Jonge et al., 2019). Contrary to predators, who may kill their prey and then use it as a source of nutrients, bacteriophages cannot use any resources from a dead organism despite the fact that they can kill bacterial cells. Bacteriophages should therefore be regarded as parasites rather than predators in accordance with biological definitions and of ecological interactions between various types of life (Węgrzyn, 2022). Bacteriophages are thought not to be harmful to humans (Principi et al., 2019). Since they can be found in all environments where bacteria can live, such as water, plants, and food, they are harboring within human bodies (Grose and Casjens, 2014). Phages have also been acknowledged as significant elements of the human natural microbiome (Zuppi et al., 2022). In the large intestine, phage virions range between 10^8^ to 8 × 10^10^/g of feces (Kim et al., 2011; Hoyles et al., 2014). Gut viral genomes are primarily composed of phages (97.7%), followed by eukaryotic (2.1%), and archaeal viruses (0.1%; Gregory et al., 2020). The whole viral community in the human gut, known as the virome, is dominated by the bacteriophage population (also referred to as the phageome; Spencer et al., 2022). Although the knowledge regarding the human phageome is constantly broadening, little is currently known about the normal chicken gastrointestinal phageome (Manrique et al., 2016).

### 2.2. Taxonomy

Due to the absence of universally conserved marker genes in bacteriophages, their taxonomy is complicated, and most phages remain unclassified and poorly characterized. The classification of bacteriophages is regularly updated and approved by the International Committee on the Taxonomy of Viruses (ICTV; Table 1), based on host range, size, structure and morphology, nucleic acid, and genomic similarity (Lefkowitz et al., 2018). The majority of phages known to date belong to the Caudovirales order (also known as the tailed phages), which have (ds) DNA with a size range of 18–500 kilobase pairs (kbp; Ackermann, 2007). According to the recent taxonomy update of the ICTV in August 2022, the phage classification was modified, and the morphology-based families Podovirus, Myovirus, and Siphovirus were abolished along with the removal of the order Caudovirales, which was replaced by a new class called “Caudoviricetes.” Currently, there are 14 families divided among four orders (*Crassvirales*, *Kirjokansivirales*, *Thumleimavirales*, and *Methanobavirales*) in the class Caudoviricetes. A total of 33 additional families have been established but have not yet been allocated to an order, along with 37 subfamilies and 631 genera that have yet to be classified at the family or order level (Turner et al., 2023).

**Table 1:** Current taxonomy of the class *Caudoviricetes* following International Committee on Taxonomy of Viruses (ICTV).

Order	Family	Subfamily	Genus	Species
*Crassvirales*	Crevaviridae	2	3	4
	Intestiviridae	3	11	18
	Steigviridae	1	12	15
	Suoliviridae	5	16	36
*Kirjokansivirales*	Graaviviridae		2	2
	Haloferuviridae		3	3
	Pyrstoviridae		1	1
	Shortaselviridae		1	1
*Methanobavirales*	Anaerodiviridae		1	1
	Leisingerviridae		1	1
*Thumleimavirales*	Druskaviridae		2	2
	Hafunaviridae		4	10
	Halomagnusviridae		1	1
	Soleiviridae		1	1
Unidentified	33	11	59	96
	Unidentified	37	631	2060

ICTV taxonomy (https://talk.ictvonline.org/taxonomy/).

### 2.3. Development cycles

**Figure 2:**
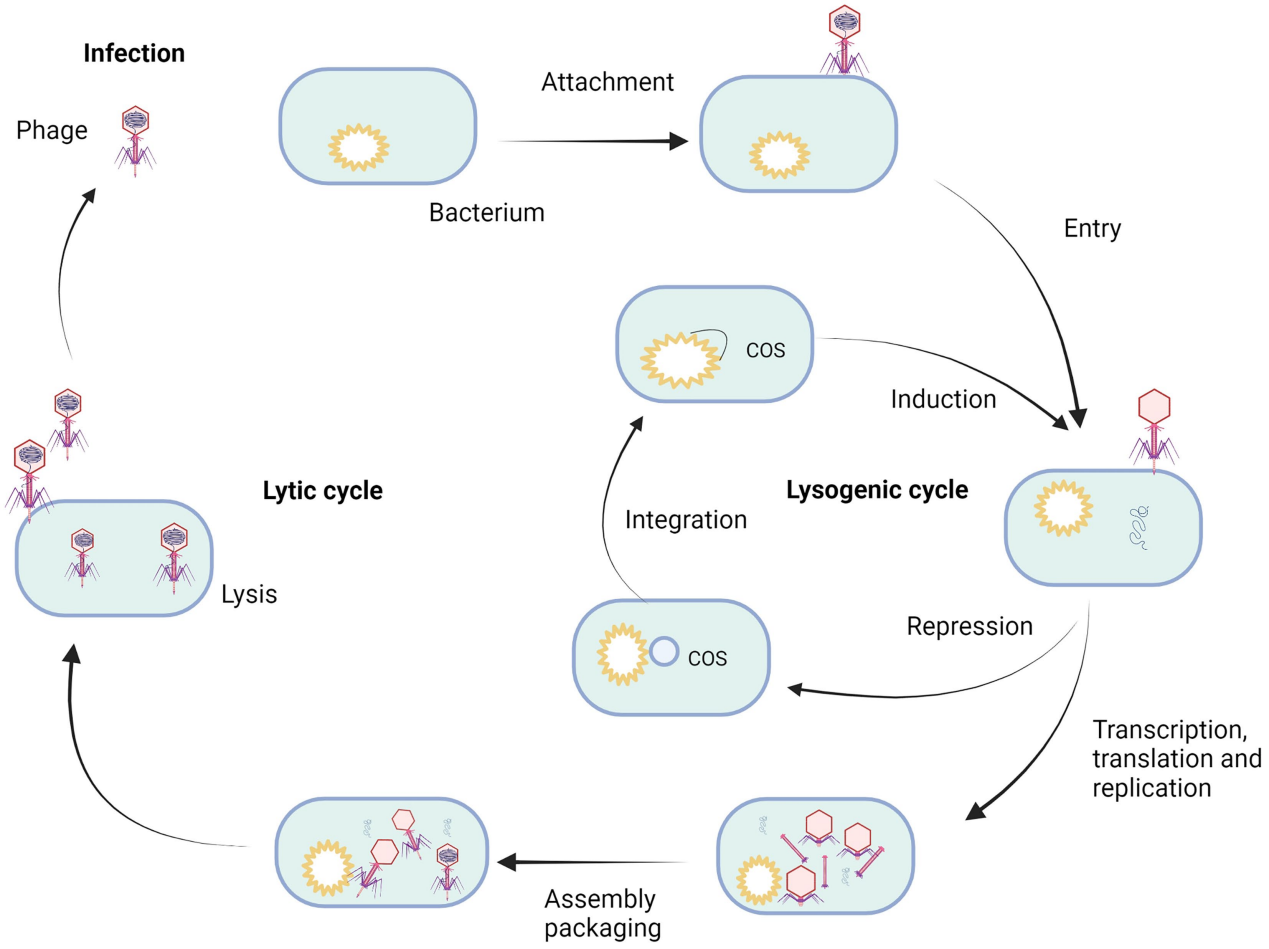
Life cycle of bacteriophage. Created with BioRender.com.

The biology of bacteriophages has been exhaustively studied in the century since their discovery. Two major bacteriophage classes have been described, lytic and temperate ones (Oppenheim et al., 2005). These differ in the way they interact with their bacterial host (Figure 2; Campbell, 2003). A lytic or virulent bacteriophage follows a strict lytic growth cycle, including the bacteriophage particle’s attachment to the bacterial host, injection of the bacteriophage genome into the bacterial host, replication (amplification of the bacteriophage genome), transcription/translation (genes expression of bacteriophage specific proteins), assembly of new bacteriophage particles, lysis (destruction of the bacterial cell wall that coincides with the death of the bacterial host), and release of the bacteriophage progeny into the environment, where they strive to find a new bacterial host to repeat the process (Oflaherty et al., 2009; Fortier and Sekulovic, 2013). The key stage distinguishing the lytic cycle from the lysogenic one is the lysis of bacterial cells (Huff et al., 2009; Fortier and Sekulovic, 2013). The protein involved in this process is holin, which opens gaps in the cytoplasmic membrane and enables the phage-encoded endolysin (also known as lysin) to enter and hydrolyze the peptidoglycan layer. This results in cell lysis and the release of progeny phages that can infect further bacterial cells (Fortier and Sekulovic, 2013). The total cycle can last from 20 min up to 2 h.

Temperate bacteriophages also have the ability to follow a lytic growth cycle, but they have also developed an alternative multiplication strategy that is not lethal for the bacterial host. The lysogenic growth cycle involves the connection of the bacteriophage particle to the bacterial host, injection of the bacteriophage genome into the bacterial host (Chen et al., 2020), repression of all bacteriophage functions that would lead to lytic growth, integration of the bacteriophage genome into the genome of the host bacterium, passive amplification of the bacteriophage genome whenever the host genome amplifies. Every bacterial daughter cell inheriting an integrated bacteriophage genome is called a prophage in this stage (Cieślik et al., 2021). This symbiotic relationship between prophage and the bacterial host can remain stable for generations. However, under stress conditions, the prophage can be induced. The repression of all the bacteriophage functions that would lead to lytic growth is released, the prophage genome is cut out of the bacterial genome, and a lytic growth cycle follows, leading to the death of the bacterial host cell and the release of progeny bacteriophages (Howard-Varona et al., 2017).

### 2.4. Antibacterial activity of bacteriophages

Compared to the widely used broad-spectrum antibiotics, bacteriophages are far more specialized. It should be highlighted that antibiotic treatment affects the normal gut microbiota in addition to killing pathogenic bacteria, which can result in dysbiosis, immunosuppression, and subsequent secondary infections (Lin et al., 2017). While polyvalent phages can attack several (two or more) bacterial species, monovalent phages are unique to one type of bacterial species. Bacteriophages that target Gram-positive bacteria are not effective against Gram-negative bacteria (Żbikowska et al., 2020). As it was mentioned, endolysins, also referred to as phage lysins or hydrolases, are produced by bacteria-eating phages and are essential for the internal lysis of the bacterial cell wall at the end of the lytic cycle (Schmelcher et al., 2012). Furthermore, because they may dissolve the peptidoglycan when applied externally to the bacterial cell, bacteriophage endolysins may serve as novel antibacterial agents (Mirski et al., 2019; Grabowski et al., 2021).

Since Gram-positive bacteria do not have an outer membrane like Gram-negative bacteria, exogenous endolysin activity is highly powerful against them. Briefly, Gram-negative bacteria are surrounded by a thin peptidoglycan cell wall, which itself is surrounded by an outer membrane containing lipopolysaccharides. Gram-positive bacteria lack an outer membrane but are surrounded by layers of peptidoglycan, many times thicker than are found in the Gram-negative ones (Silhavy et al., 2010). Gram-negative bacteria are challenging to lyse as endolysin cannot access the peptidoglycan because of the outer membrane. Nevertheless, using bacteriophage proteins is a promising approach to novel antibacterial strategies.

### 2.5. Advantages and disadvantages of bacteriophages

The most alluring quality of bacteriophages is their capacity to destroy only the targeted bacteria, which is known as their specificity of action. They have a very limited range of activity, avoiding the main issue directly related to the use of antibiotics, which is the impact on the entire microbiome with the eradication of potentially helpful bacteria and the proliferation of secondary pathogens (Domingo-Calap and Delgado-Martínez, 2018). Bacteriophages are believed to be substantially safer and more tolerable because they exclusively multiply in the particular bacterial cells that they are intended to infect (Kakasis and Panitsa, 2019). Despite all the advantages, the use of bacteriophages has its limitations. It is challenging to prepare bacteriophages for therapeutic application, and not all issues directly related to the biology of these viruses have been resolved (Lin et al., 2022).

The great specificity of bacteriophages can also be seen as a disadvantage because their cleavage spectrum may be too narrow. Bacteriophages often only affect a small number of bacterial species or genera, making it impossible for them to specifically target all pathogenic strains of a particular bacterial species (Hyman and Abedon, 2010). Although bacteriophages are helpful in treating illnesses brought on by a single bacterium, infections caused by a number of harmful bacteria are frequently observed in clinical cases (Gill and Hyman, 2010). As a result, it is challenging for particular bacteriophages to achieve the intended therapeutic impact (Gill and Hyman, 2010). To be used most effectively in therapy, phages can be combined as “cocktails” to broaden their host range coverage, improve killing efficiency or limit the development of phage resistance (Chan et al., 2013). Many phage cocktails have been designed against *Salmonella* and their efficacy has been tested in challenge studies both in swine and poultry (Martinez et al., 2019).

Another limitation of phage therapy is related to using of temperate phages. The lysogenic phenomenon is characterized by the fact that some phages are unable to lyse the host bacteria and prevent other phages from lysing their host bacterium following integration. When a virus exhibits lysogenicity, the host DNA and viral genome multiply together, either in a free plasmid-like condition or after integration into the bacterial chromosome (Carascal et al., 2022). Thus, using lysogenic phages explicitly translate into the inefficiency of the treatment. Moreover, the specificity of the lysogenic cycle may contribute to the increased risk of the distribution of harmful genes in the environment. The fact that bacteriophages in the lysogenic condition can also spread toxins and genes for antibiotic resistance to bacteria may pose a significant threat to public health.

Since 2011, the phages have been classified as drugs in the United States and as medicinal products in European Union (EU; Guo et al., 2020; Naureen et al., 2020). However, there are a number of factors that create regulatory barriers for to the global manufacture and use of phages as substitutes for or at the very least as a supplementary treatment option over conventional antibiotics. There is a lack of understanding concerning phage therapy since there is a paucity of evidence from clinical trials that were conducted in accordance with national and international ethical standards (Guo et al., 2020; Naureen et al., 2020).

## 3. Phage therapy of bacterial infections and zoonotic agents in poultry

Many studies have focused on the effectiveness of bacteriophages in reducing bacterial count in poultry. Bacteriophages have been used to protect animals from infections caused by pathogens, which have a significant influence on public health like *Salmonella enterica subspecies enterica* serovar Enteritidis, *S. enterica subspecies enterica* serovar Typhimurium, *C. jejuni*, *E. coli*, *Listeria monocytogenes*, and methicillin-resistant *S. aureus* (MRSA; Tiwari et al., 2011; Wernicki et al., 2017; Moye et al., 2018).

### 3.1. *Salmonella* spp.

Numerous *Salmonella* serovars prevalent in chicken are the main cause of human food-borne illness. Bacteria can colonize a wide range of animals, serving as reservoirs and vectors for the transmission of these infections to both animal and human populations. *S. enterica* serovars are still among the world’s most prevalent food-borne pathogens (Fatima et al., 2022). Public concern over antibiotic-resistant strains, especially among zoonotic pathogens like *Salmonella*, has driven the poultry sector to identify alternative control methods (Boyle et al., 2007).

*Salmonella* Enteritidis causes the majority of human salmonellosis cases in the EU, and the proportion of human cases attributable to this serovar remained unchanged from 2017 to 2018 (20.1 cases per 100,000 population; EFSA, 2018). Similar to previous years, eggs and egg products were the primary sources of *Salmonella* food-borne illnesses in 2018 (EFSA BIOHAZ Panel (EFSA Panel on Biological Hazards), 2019). Poultry meat contained the greatest proportion (7.6%) of *Salmonella*-positive samples in meals (EFSA and ECDC, 2021).

Reducing microbial contamination during poultry production is vital because many of the resulting food-borne diseases are linked to poultry products (De Cesare, 2018; Ehuwa et al., 2021). Andreatti Filho et al. (2007) noted that broiler chicks may experience a temporary decrease in *Salmonella* Enteritidis recovery after receiving bacteriophage mixes, but 48 h later, there was no difference between treated and untreated groups. Moreover, compared to bacteriophages alone, the bacteriophage cocktail plus a probiotic culture had no impact on the quantity of *Salmonella* Enteritidis. In-depth research has been carried out, investigating potentials of bacteriophages in chickens for *Salmonella*-related diseases in addition to suppressing paratyphoid *Salmonella* (Wernicki et al., 2017). Berchieri et al. (1991) used bacteriophages (*S.* Typhimurium strains F98 [phage type 14), Beauville (phage type 40), and 1,116 (phage type 141)] at a dose of 10^12^ plaque forming units (PFUs)/mL to treat birds challenged with *S*. Typhimurium and discovered that the mortality rate associated with *S*. Typhimurium could be decreased to 20% compared with 56% in the untreated group. However, *S.* Typhimurium was not eradicated and recovered to its previous levels 6 h after treatment. Furthermore, the bacteriophages did not survive in the gastrointestinal tract if *Salmonella* was present. Generally, bacteriophages only lasted as long as they were orally administered as a feed additive. The authors concluded that bacteriophages must be supplied in large quantities and immediately after infection with *S*. Typhimurium in order to be effective (Hurley et al., 2008) found that the bacteriophages may kill *S.* Typhimurium through an excess of bacteriophage administration. With phage treatment but without the *Salmonella* challenge, the chickens had a decreased rate of mortality.

The initial findings from the extensive usage of *Salmonella* phages in a poultry production system were released in 2019 (Clavijo et al., 2019) and concerned the first commercial bacteriophage product, Biotector S1® (CJ CheilJedang Research Institute of Biotechnology, Seoul, South Korea), which can be used as an additive in feed to prevent *S. enterica* subspecies *enterica* serovar Pullorum (*S*. Pullorum) and *S. enterica* subspecies *enterica* serovar Gallinarum (*S.* Gallinarum) in poultry. In commercial broilers (five-week-old Ross), the experimentally treated groups that received Biotector S1® at varied concentrations in feed (5 × 10^7^, 1 × 10^8^, and 2 × 10^8^ PFUs/kg) exhibited a lower mortality rate (73%) following challenge compared with the control group. There were no observable differences in mortality among the experimental groups (2.78, 3.13, 3.13%). The mortality rate (45%) in the group of broiler breeders (67-week-old Ross) getting bacteriophages (1 × 10^6^ PFUs/kg) was lowered by 53% when compared to the non-phage treated control (85%) after challenge. The layers (six-week-old Lohmann Brown) that received the same dose (1 × 10^6^ PFUs/kg) prior to the challenge showed the greatest reduction in mortality (by 86%). After the challenge, mortality in the control group decreased to 35%. Egg production increased by 3% (trial 90.6%, control 87.5%), and egg mass (g/day/bird) increased by 2.4% (trial 59.2%, control 56.8%) in the Hy-Line Brown layers when exposed to phage (1 × 10^8^ PFUs/kg; Kim et al., 2012).

The use of the bacteriophage mixture (SalmoFREE®) in drinking water proved to be safe several times. Neither the chickens’ behavior nor the production metrics were impacted. At the end of the fattening period (day 33), the percentage of *Salmonella* in cloacal swabs was zero in comparison with the control henhouses where the *Salmonella* were still detected.

Another feed additive for birds is called Bafasal® (Proteon Pharmaceuticals, Poland), and is given by water. In field applications, Bafasal® was shown to have a significant impact on food safety by reducing *Salmonella* levels up to 200 times while also improving the feed conversion ratio (FCR) and lowering deaths of animals. Despite not requiring a waiting period before eating meat or eggs, Bafasal® treatment does have both a preventative and a post-infection interventional impact (Wójcik et al., 2015; EFSA, 2021).

The study by Lorenzo-Rebenaque et al. (2022) showed that preventive therapy with *Salmonella* phage minimally alters the cecal microbiota but significantly impacts cecal microbiota metabolites regardless of the route of administration. The phage vB_SalP_LDW16 (family Siphoviridae in the order *Caudoviridae*) is a lytic phage with a broad host range that may be utilized as a substitute in livestock husbandry to prevent and treat chicken salmonellosis (Cao et al., 2022). Diverse serovars of *Salmonella* were recovered in the broiler production chain in that study, while the isolates presenting ciprofloxacin-resistant *Salmonella* were as high as 29.4%. Overall, *Salmonella* phages showed high lysis ability against these ciprofloxacin-resistant *Salmonella* isolates, suggesting the potential application of phage-based treatments or biocontrol in the broiler production chain (Pelyuntha et al., 2022). Grabowski et al. (2022) indicated for the first time that cocktail of phages targeting *Salmonella* is not only effective but also can be used in veterinary practices without disturbing immune homeostasis, expressed as cytokine imbalance, disturbed percentage of key immune cell subpopulations, and stress axis hyperactivity. Kosznik-Kwaśnicka et al. (2022a) reported that phage therapy against *S*. Typhimurium infection in chickens appeared as effective as antibiotic therapy (with either enrofloxacin or colistin), but was less invasive than the use of antibiotics as fewer changes in the microbiome were observed. Li et al. (2022) showed promise for the use of a combination of the GRNsp6, GRNsp8, and GRNsp51 phages as an efficient antimicrobial treatment agent against multidrug-resistant *Salmonella* in animal production to reduce infections by various zoonotic *Salmonella* species. Kosznik-Kwaśnicka et al. (2022b) demonstrated high efficacy and acceptable safety profiles of phage therapy against *S. enterica* strains using vB_SenM-2 and vB_Sen-TO17 phages (both alone and in a cocktail). These results open the possibility for phage treatment trials in poultry and, indeed, these phages might serve as a basis for future phage therapy in poultry farming.

Phages may also be effective in the pre-treatment model. For instance, the most effective method for biocontrol of *Salmonella* strains was reported to be pre-treatment with *Salmonella* phage STP4-a (Li et al., 2020). A total of 7 days before challenging two-week-old layer hens with 8 log_10_ colony forming units (CFUs) of *S.* Typhimurium, the authors pre-treated the chicks with 9 log_10_ PFUs/g phage STP4-a. In fecal samples, results showed a 3–5 log_10_ CFUs bacterial reduction within 30 min, and colonies were not found during the course of the 14-day trial period. Furthermore, Fiorentin et al. (2005) demonstrated that the *S.* Enteritidis loads were decreased by 3.5 log_10_ CFUs/g following a single-dose oral injection of a high-tittered (10^11^ PFUs) phage cocktail (CNPSA1, CNPSA3, and CNPSA4).

According to Wernicki et al. (2017), phage therapy may also be helpful in reducing horizontal transmission within poultry flocks. Commercial laying hens fed a phage-supplemented diet experienced a drop in mortality rate from 30 to 5% after coming into contact with flocks infected with *S.* Gallinarum (Lim et al., 2011). It was hypothesized that the effectiveness of phage therapy could be even improved by utilizing a high phage titer to reduce *Salmonella* colonization through passive transmission.

### 3.2. *Campylobacter* spp.

*Campylobacter* spp. are common in many settings, but they favor the gut of birds where they live as commensals (Humphrey et al., 2014). On poultry farms, *Campylobacter* is rarely detected in birds younger than 2–3 weeks of age (Zhang and Sahin, 2020). Despite being *Campylobacter* carriers, chickens rarely show any clinical symptoms or lesions. According to Sahin et al. (2015), there are wide variations in the incidence of *Campylobacter* spp. among poultry flocks, ranging from 2 to 100%. According to study findings, the prevalence of *Campylobacter* spp. among broiler flocks and in chickens at the time of slaughter varies from 42.5 to 100% (EFSA, 2017). The *Campylobacter* spp. incidence was reported to be 40% in broilers in 2021 in the EU Member States and three non-EU Member States by the European Food Safety Authority (EFSA) and the European Centre for Disease Prevention and Control (ECDC; EFSA and ECDC, 2022). Additionally, efforts to reduce *Campylobacter* prevalence are urgently required due to increased reports about the bacterial pathogenicity and antibiotic resistance to fluoroquinolones, tetracycline, erythromycin, and gentamicin (Nowaczek et al., 2019).

The prevalence of *Campylobacter*-specific bacteriophages in poultry is typically low, and the majority of them belong to the *Myoviridae* family and infrequently to the *Siphoviridae* family (Firlieyanti et al., 2016; Nowaczek et al., 2019). A recent experimental study has shown the efficacy of phage treatment in suppressing *Campylobacter* colonization in chickens, and so lowering the danger of *Campylobacter* entering the food chain. Pre-harvest phage therapies have boosted efficiency against *Campylobacter* loads in the feces and intestinal contents of experimentally infected chickens without having any negative impacts on the health of the birds, according to studies. For instance, Chinivasagam et al. (2020) observed a significant bacterial reduction of 1–3 log_10_ CFUs/g in the ceca 28 h post-treatment when they orally administered a mixture of *Campylobacter* phages to 47-day-old broiler chickens.

According to modeling approaches, bacteriophage control to lower *C. jejuni* levels in chickens could lessen human exposure and sickness brought on by eating contaminated poultry products (Richards et al., 2019). A decrease was seen in 20-day-old chicks treated with phage CP14 (5 × 10^8^ PFUs) throughout a 31-day period, compared with the negative control (Hammerl et al., 2014). According to Carvalho et al. (2010), after 2 days of the administration, the presence of *C. jejuni* and *C. coli* in poultry feces was reduced by about 2 log_10_ CFUs/g after oral gavage and in-feed application of a three-phage cocktail. However, it has been claimed that chickens treated with phages have recovered some resistant bacterial phenotypes, while *Campylobacter* reduction by phages was not impeded (Loc Carrillo et al., 2005; Carvalho et al., 2010; Fischer et al., 2013). The critical elements for successful phage therapy to treat *Campylobacter* are the right phage selection and optimization of the delivered technique and dosage, as shown by this previous research study on chickens (Loc Carrillo et al., 2005). Field trials have underlined the potential of *Campylobacter*-specific bacteriophages in commercial settings but also indicated issues regarding standardization and reproducibility (Kittler et al., 2013; Chinivasagam et al., 2020).

There are currently no commercially available phage products against *Campylobacter* spp. despite the evident need to deploy innovative methods aimed at lowering the rate of infections caused by these bacteria (Richards et al., 2019). This might be because *Campylobacter* phages differ from the majority of other lytic phages in some ways, making it challenging to use them. The optimal approaches for phage isolation, propagation, and purification are primarily responsible for the difficulties encountered in the creation of safe *Campylobacter* phage cocktails. Even though they are genetically extremely similar, *Campylobacter* phages within groups differ in a variety of ways (such as host range, lytic activity, and kinetics), which makes it challenging to choose the right phage candidates for application. Recently, Steffan et al. (2022) showed that greater use of sophisticated selection schemes combining techniques like planktonic death assays (PKAs), which might be paired with standardized analysis methodologies, could be beneficial for the creation of phage cocktails in general. The current study outlines a continuous workflow for determining host range and efficiency of plating (EOP) value in conjunction with a qPCR-based phage group identification and a PKA assay, which allowed us to evaluate phage cocktails with a sophisticated analysis framework that was based in part on the virulence index. It was discovered that a combination of the group II phage vB CcM-LmqsCP218-2c2 and the group III phage vB CjM-LmqsCP1-1 showed promise for use against *C. coli* and *jejuni* (Steffan et al., 2022).

### 3.3. Escherichia coli

*Escherichia coli* is one of the main health threats in the poultry industry worldwide. It causes serious health problems in animals, including airsacculitis, colisepticemia, coligranuloma, enteritis, omphalitis, orchitis, osteomyelitis, panophthalmitis, peritonitis, salpingitis, septicemia, and swollen-head syndrome (Nolan et al., 2020). Taken together, *E. coli* infections result in severe economic losses.

The potential for treating *E. coli* infections in chickens with bacterial viruses has been examined in a number of studies. Lytic bacteriophages for *E. coli* were used by Barrow et al. (1998) to reduce the illness and mortality of infected poultry. When hens were administered 10^6^–10^8^ PFUs of bacteriophages at the same time as intramuscular *E. coli* infection, the death rate was reduced by 100%. This study also demonstrated that when host bacteria are present in both the blood and the brain, bacteriophages can cross the blood–brain barrier and multiply. Bacteriophages were used by Huff et al. (2003) to treat chicken airsacculitis caused by *E. coli*. By injecting bacteriophage along with the bacterial challenge inoculum into the thoracic air sac, significant effectiveness was reached. However, administering the same bacteriophages through drinking water was not effective in preventing the onset of the disease. Additionally, it was shown that bacteriophage aerosol therapy, followed by an *E. coli* challenge the following day, 2 days later, or 3 days later, reduced death linked to respiratory illness (Huff et al., 2002). Thus, that study demonstrated the respiratory system’s capacity for bacteriophage prevention. Prophylaxis without continuing delivery or knowing that an animal has been exposed, however, could be challenging given the data that bacteriophages often do not survive in the absence of an adequate host (Fiorentin et al., 2005; Oot et al., 2007; Hurley et al., 2008). Commercial flocks of chicken cannot be treated with injectables on an individual basis, but very pricey breeder flocks might be worth the time and money. However, these successes might not always result in efficient intestinal treatments (Tiwari et al., 2011). Due to host-associated pressure against pathogen infections, systemic bacteriophage therapy may be effective. When bacteriophages are used to treat systemic or tissue-associated infections in these situations, a simple reduction in infection load of 90% or more could have a major impact on mortality as well as the course and severity of the disease. Recently, Wang et al. (2022) found that phage GN06 had significant inhibition of avian pathogenic *E. coli* both within the liquid medium and in biofilm formation. A wild pigeon’s droppings included United Arab Emirates MI-01, a monovalent bacteriophage with lytic activity against *E. coli* O157: H7. Given that wild birds have *E. coli* O157:H7 phages, it is likely that they are carrying pathogenic *E. coli* O157:H7 (Sultan-Alolama et al., 2022). Jiang et al. (2022) isolated phages flora and kanamycin sulphate (KM18) targeting *E. coli*. Compared to KM18, phage flora has a wider lytic spectrum. In increased *E. coli* cultures, phage flora also outperformed kanamycin sulfate in terms of its antibiofilm effects. In minimal *E. coli* cultures, the phage flora and kanamycin sulphate together demonstrated superior antibiofilm actions over flora or kanamycin sulphate alone.

### 3.4. *Staphylococcus* spp.

Staphylococci, including *S. aureus*, are common in the immediate environment of poultry and are typical occupants of healthy birds’ skin and mucous membranes (Andreasen, 2020). *S. aureus* infections are a global issue in the production of chickens and turkeys, and they result in economic losses due to lower output, mortality, and carcass condemnation at slaughter. Arthritis, synovitis, chondronecrosis, osteomyelitis, gangrenous dermatitis, subdermal abscesses (bumblefoot), green liver-osteomyelitis complex, and septicemia are conditions caused by *S. aureus* infections in turkeys (Andreasen, 2013, 2020). Particular concerns may also be raised if MRSA is found in poultry meat (Feßler et al., 2011).

The phages attacking the genus *Staphylococcus* are called staphylophages. Based on the genome size, staphylophages were grouped into three classes: class I-*Podoviridae* (most of them having the smallest genome), class II-*Siphoviridae* (intermediate genome size), and class III-*Myoviridae* (the largest genome; Leskinen et al., 2017). *S. aureus* strains found in broiler chickens and turkeys gave rise to bacteriophages that belonged to the *Siphoviridae* family of the *Caudovirales* order. They had an icosahedral head, a long, thin, flexible tail that was not contractile, and a double-stranded DNA structure. They belonged to the three serogroups A, B, and F with the subgroups Fa and Fb and had a high lytic impact against *Staphylococcus* strains as well as other bacteria. Despite having excellent *S. aureus* selectivity, certain bacteriophages had enterotoxigenic genes, making them unsuitable for phage therapy (Marek et al., 2019). Currently, there are no phage formulations intended to both prevent and treat infections brought on by *S. aureus* in poultry. Additionally, to date, there are no experimental data regarding phage therapy of staphylococcal infections in poultry.

## 4. Enhancing the absorption of nutrients and performance of poultry

The development of natural compounds as antibiotic alternatives has been the subject of several studies. These can be added to feed to help poultry flocks achieve better growth, performance, health, immunity, and gut microbiome (Islam et al., 2020). However, there are few studies that are available, and those that are mixed results regarding the use or supplementation of phages to improve chicken growth performance and nutrient digestibility. For instance, Upadhaya et al. (2021) found that feeding broiler chickens commercial products containing phages against different bacteria at the levels of 0.05 and 0.1% had no effect on their feed intake, FCR, or apparent total tract digestibility. However, higher titers of phages linearly enhanced body weight (BW) gain at days 1–7 and 22–35. The phage supplementation, according to that study, encouraged the growth of *Lactobacillus* and other advantageous bacteria in the stomach of broilers. In a related study by Kim et al. (2013), hens fed feed supplemented with phages at 0.1 and 0.2% demonstrated higher BW gain and decreased FCR than those supplemented with 0.05%. On the other hand, Wang et al. (2013) revealed that adding 0.5 g/kg of bacteriophage to the diet enhanced liver weight and feed efficiency in the beginning phase without changing the characteristics of the breast muscle. The same supplementation also helped broilers by preventing the shedding of pathogens from excreta. Higher phage dosage was required to enhance poultry performance by lowering intestinal *S.* Typhimurium and *S.* Enteritidi*s* (Atterbury et al., 2007). According to Zhao et al. (2012), adding phages (against a mixture of *S.* Typhimurium, *S.* Gallinarum, and *S.* Enteritidis) to the diet (0.035% or 0.05%) improved egg production and egg quality. Overall, further research is needed to examine how phages affect growth performance and nutrient digestibility as well as to create phage products that are affordable to employ in chicken production systems.

## 5. Phage therapy failures

The authors believe there are several unpublished examples of failures to treat enteric *Enterobacteriaceae* infections, given that experimental failures are typically not documented and that the cause of failure is frequently unknown. However, some of these errors or half-measures have been recorded and are described below. *Salmonella* was chosen as an example for these serious discussions.

Hurley et al. (2008) stated that to better understand the biological aspects of the luminal ecology, *Salmonella*, and bacteriophages as well as how they interact within the gastrointestinal tract, bacteriophage SP6 was utilized to predict parameters for treating *Salmonella*-infected hens. *Salmonella* resistance to bacteriophages, variable growth rates, and feed and water intake were assessed. The results of these *in silico* testing were taken into account while creating an *in vivo* challenge. *Salmonella* was discovered at levels that did not differ from the control groups after bacteriophage therapy. In fact, 50% of the *Salmonella* isolates from a treated group were resistant to bacteriophage SP6 on day 29 (Hurley et al., 2008). Furthermore, many of the *Salmonella* isolates cultured from other samples of bacteriophage-treated birds showed at least partial resistance to bacteriophages, with only partially clear plaques forming on soft agar overlays when the *Salmonella* isolate was susceptible, and clear plaques routinely formed on soft agar overlays prior to bacteriophage treatment. Furthermore, despite ongoing high levels of *Salmonella* recovery within the cecum, the investigators observed a continuous decline in bacteriophage excretion (Hurley et al., 2008).

This evidence is consistent with the findings of Fiorentin et al. (2005), who observed that even while bacteriophage levels had decreased to undetectable levels 21 days following inoculation, *Salmonella* was still detectable. In another study, turkeys treated with bacteriophages recovered to higher levels of *Salmonella* 48 h after initially declining at 6, 12, and 24 h (Higgins et al., 2007). These bacteriophages were chosen for their capacity to withstand low pH, to simulate passage through the ventriculus of chickens, and to be administered with Mg (OH)_2_ to assist bacteriophage attachment to bacterial cell walls (Eisenstark, 1967). The authors also reported that bacteriophage resistance was widespread across all cultures. Resistance to bacteriophages selected against *Salmonella* isolates arises swiftly, typically in a single passage, as documented in some studies (Fiorentin et al., 2005; Bastías et al., 2010).

Even when the bacteriophage cocktail was used repeatedly or continuously, rebound levels were similar to the controls within 48 h after experimental *Salmonella* Enteritidis infections in chickens challenged with the bacteria. This was true even when the bacteriophage cocktail contained 71 different bacteriophages against *Salmonella* (Higgins et al., 2007, 2008).

In order to potentially deliver more bacteriophages to the infection site, several attempts were made to protect the bacteriophage cocktail *via* the upper gastrointestinal tract (Bielke et al., 2007a,b). Although pre-treating infected poultry with antacid products intended to lessen the proventriculus’ acidity was effective in enhancing the number of delivered bacteriophages that successfully passed into the intestinal tract, this treatment had no positive effects on how *Salmonella* Enteritidis was treated with bacteriophages (Higgins et al., 2007).

Alternative non-pathogenic bacteriophage hosts may be able to “carry” bacteriophages through the gastrointestinal tract and, with the continual food supply of the non-infected substitute host bacterium, provide a method of amplification inside the host’s gut (Bielke et al., 2007a). Bielke et al. (2007a,b) revealed that some bacteriophages recovered from a *Salmonella* Enteritidis can be chosen as non-pathogenic substitute hosts. This method was also used to develop a putative “Trojan Horse” model for bacteriophage protection through the upper gastrointestinal tract, which may have served as a pathway for enteric amplification of any bacteriophages that survived. But neither the “Trojan Horse” technique nor the continued feeding of the substitute host bacteria as a source of enteric amplification were successful in reducing enteric *Salmonella* infections more than a temporary amount.

## 6. Limitations of phage application in the poultry industry

Bacteriophage resistance is a critical component of therapy that must be overcome before bacteriophages can truly be used as an antibiotic alternative. Bacterial growth times are usually short enough that mutants with bacteriophage resistance can emerge within hours (Lowbury and Hood, 1953; Higgins et al., 2007). One possible solution to this problem is to administer numerous bacteriophage isolates for treatment (Bielke et al., 2007a,b). Kittler et al. (2014) observed a possibility that phase variation occurred and that it may have had pleiotropic effects on gamma-glutamyl transpeptidase (GTT) activity. Additionally, two distinct mechanisms might have mediated resistance to various cocktail phages and affected various metabolic characteristics of the isolates.

Food and meat processing plants are a good alternative. As live animals are brought to a processing facility, the germs are unlikely to have been exposed to the bacteriophages used to cure the infection. This considerably enhances the likelihood of success. Higgins et al. (2005) successfully treated turkey carcasses at a processing facility using bacteriophages specific to the *Salmonella* strains that contaminated them. When either an autogenous bacteriophage treatment targeted the specific *Salmonella* strain infecting the turkeys, or a mixture of nine wide host-range *Salmonella*-targeting bacteriophages was applied, this method was effective.

It is likely that choosing a number of bacteriophages with the same target cell phenotype leads to the choice of bacteriophages with the same adhesion, penetration, replication, and release processes (Lorenzo-Rebenaque et al., 2021). The target cell’s capacity to shift phenotype may be severely constrained when new bacteriophages are isolated for effectiveness against sequentially resistant isolates of the target bacteria and combined for administration as a cocktail, leading to a much larger proportion of target cell reduction and raising the likelihood of elimination or cure. During phage therapy, it is important to record the bacteria’s susceptibility to the phage as well as the stability and effectiveness of the phage (Żbikowska et al., 2020).

The effectiveness of bacteriophages depends on their ability to replicate well and survive in specific environments. It would appear prudent to enhance procedures for phage selection and separation from the host environment in order to reach their maximum potency (Batinovic et al., 2019). Samples with bacteriophages can be purified using a number of well-described techniques, both from bacterial cultures and environmental samples (Bonilla and Barr, 2018; Deng et al., 2019). As they are easy, cheap, and well-characterized processes, filtering, polyethylene glycol precipitation, and cesium chloride gradient centrifugation are among the most often utilized techniques. However, it has been noted that some phages are adversely affected by these specific purification techniques themselves (Kleiner et al., 2015).

Phages cannot be pyrogenic or allergic (sterility tests, lack of residual endotoxins). It is commonly acknowledged that bacteriophages’ ability to produce specific antibody humoral responses and immunogenicity may have an impact on phage therapy in both humans and other species, such as chickens (Cisek et al., 2017; Majewska et al., 2019). Despite the fact that during the initial oral safety trial in humans, no anti-phage antibodies were found (Bruttin and Brüssow, 2005), later studies showed that treatment may result in different quantities of antibodies, which might not always affect the course of treatment (Żaczek et al., 2016). According to other studies, the phage type and administration route both affect the anti-phage activity in human sera (Gembara and Dąbrowska, 2021; Łusiak-Szelachowska et al., 2022).

It is unknown how the immune system of the host and the phage interact. Phages should be tested for their resistance to antibody neutralization, according to a recent study (Naghizadeh et al., 2019). The development of modern technical solutions, such as phage encapsulation, has increased phage safety when added directly to food products and animal feed, as the immune reaction of the host relies on the administration route with reduced impact after oral application (Choińska-Pulit et al., 2015).

## 7. Regulatory status of bacteriophage products

One of the most significant limiting factors to the widespread use of bacterial viruses is the regulatory status of bacteriophage products. In the United States, bacteriophage-based products against a variety of pathogens have received the GRAS (Generally recognized as safe) status from the Food and Drug Administration (FDA), and countries such as Switzerland, Israel, Canada, Australia, New Zealand, or Brazil, have approved bacteriophage-based products for diverse food applications. In the European Union, the EFSA evaluated a bacteriophage application against *Listeria* as being safe (EFSA, 2016). However, there is currently no regulatory pathway open to register a bacteriophage-based product, as there is no consensus on how such a product should be regulated, no matter whether the use as a feed additive, as a pre-harvest intervention or as a post-harvest application is considered. This legal uncertainty slows the development of commercially available bacteriophage products in Europe. Nevertheless, on a positive note, the European Medicinal Agency (EMA) has started to work on a concept paper on the quality, safety, and efficacy of bacteriophages as veterinary medicines (EMA/CVMP/NTWP/32862/2022). In light of the current problems with zoonotic pathogens, it can be hoped that these efforts by the EMA will break the legal ground for bacteriophage-based products in the poultry industry.

## 8. Conclusion

Although bacteriophage treatment for enteric disorders has had a great deal of success, it has not yet reached its full potential. Antibiotics usually work well against a wide range of bacterial species. Therefore, a specific selection is not necessary when using them to treat infections. Contrarily, bacteriophages typically target a single host and occasionally fail to eradicate all members of a given bacterial species. However, given the growth in antibiotic resistance, bacteriophages might be able to operate as a last line of defense when antibiotics are either not available or inefficient.

With the restriction or elimination of antibiotic use in food animals, researchers have been looking into the use of bacteriophages to manage food-borne infections. It is still necessary to address such issues as safety, specificity, and long-term effectiveness before using phages in poultry production. However, a breakthrough in this area is urgently needed due to the limited availability of innovative antimicrobial drugs and the widespread resistance among many pathogenic enteric *Enterobacteriaceae.*

## Author contributions

AAEW and AS were involved in the literature review, writing of the original draft, and in figure illustration. SB, HE-S, MA, LB, and BH were involved in the literature review. AAEW, GT-I, AS, and CV were involved in conceptualization, preparing the manuscript draft, reviewing, and editing. WE, HL, SK, and CV were involved in revising the manuscript. All authors contributed to the article and approved the submitted version.

## Funding

This Open Access publication was funded by the Deutsche Forschungsgemeinschaft (DFG, German Research Foundation) - 491094227 “Open Access Publication Funding” and the University of Veterinary Medicine Hannover, Foundation.

## Conflict of interest

HL was employed by the company PTC Phage Technology Center, Finktec Group and AS was employed by the company PerNaturam.

The remaining authors declare that the research was conducted in the absence of any commercial or financial relationships that could be construed as a potential conflict of interest.

## Publisher’s note

All claims expressed in this article are solely those of the authors and do not necessarily represent those of their affiliated organizations, or those of the publisher, the editors and the reviewers. Any product that may be evaluated in this article, or claim that may be made by its manufacturer, is not guaranteed or endorsed by the publisher.
